# Neuroleptic Malignant Syndrome (NMS) on Clozapine with a Potential Atypical Interaction with Paliperidone

**DOI:** 10.1155/2021/5584104

**Published:** 2021-04-28

**Authors:** Paras Agarwal, Adanegbe Omoruyi, Kiara Gascon Perai, Kerenza MacDaid, Andrea Burton

**Affiliations:** Department of General Medicine, Ashford & St. Peter's Hospital, Chertsey, Surrey KT16 0PZ, UK

## Abstract

Neuroleptic Malignant Syndrome (NMS) associated with the use of first-generation antipsychotics is a widely known phenomenon. This idiosyncratic reaction is less significant with the use of second-generation antipsychotics, and only a few cases in the literature exist, describing this reaction with clozapine use. While being titrated on clozapine, the patient developed major and minor criteria features of NMS as per the Diagnostic and Statistical Manual of Mental Disorders, Fifth edition (DSM-5) criteria except for fever, a core symptom which created diagnostic uncertainty. Initially, clozapine was temporarily discontinued due to his deteriorating mental and physical state. A rechallenge was considered at a much lower dose, but due to a rapid increase in his creatinine kinase (CK) levels within a 12-hour timeframe, clozapine was permanently stopped. The evidence further suggests that the presentation of NMS for patients on this medication may be different to the classical presentation, and other criteria for diagnosis are suggested, which may lower the threshold for investigating NMS for patients on clozapine.

## 1. Introduction

There are extremely few reported cases of clozapine associated NMS without fever. The diagnosis of NMS as per the DSM-5 criteria is made when a there is evidence of exposure to a dopamine antagonist, muscle rigidity, hyperthermia, and at least 2 of diaphoresis, tremor, altered level of consciousness, labile BP, tachycardia, elevated CK, or leucocytosis.

A case review in 2015 revealed that tachycardia, tachypnoea, blood pressure lability, and other autonomic symptoms were frequent and severe, possibly related to the high affinity of clozapine for adrenergic and muscarinic receptors. It was also noted that extrapyramidal symptoms (EPS) and fever were less reported symptoms for patients on clozapine [1].

If the DSM-5 criteria were applied to this patient, he would not have met the threshold for diagnosis of NMS due to the absence of fever on repeated measurements.

## 2. Patient Information

The patient is a 44-year-old male well known to mental health services with an established diagnosis of paranoid schizophrenia. An emergency admission to an inpatient psychiatric unit was warranted following concerns by community teams with regard to a deterioration in his mental health secondary to poor compliance with oral antipsychotic medication. As a result of his psychotic symptoms not controlled on various oral and injectable antipsychotics such as olanzapine, risperidone, and paliperidone, the multidisciplinary team (MDT) decided to commence him on clozapine.

This gentleman was not on any regular medications and was otherwise fit and healthy. His cardiac health, long-term blood sugar control, and body mass index (BMI) were all within normal ranges.

## 3. Clinical Findings and Timeline

On commencing clozapine at 12.5 mg once a day, with daily up titration by 25 mg from day 3, the patient developed autonomic side effects such as dizziness, hypotension, and tachycardia as early as day 2 while on a total daily dose of 25 mg. Over the next 5 days, the patient appeared increasingly withdrawn, spending more time in his bed space. He became thought disordered and was noted to be frequently drooling. The patient remained intermittently tachycardic with other vital signs within normal range. His symptoms worsened by day 10 as he developed dyskinesia, diaphoresis, mild truncal rigidity, and worsened mental state. He was tachycardic with the highest recorded heart rate as 135 beats per minute. His temperature remained <38°C at all times.

On day 10, the patient, while in a state of confusion, ran into a closed door. He was referred to the accident and emergency department for a thorough physical health evaluation as he suffered bruises to his face and to rule out intracranial injury. He was on 175 mg total daily dose of clozapine, and this was discontinued. A serum creatinine kinase (CK) level was 651 U/L (40-320). The medical team attributed this to the traumatic musculoskeletal injury. Due to the mild rise in CK, clozapine was restarted at a much lower 25 mg once daily dose, and a management plan was created to frequently monitor CK levels. The patient was transferred back to the inpatient psychiatric unit. A repeat CK level on the same evening yielded a result of 1757 U/L. An MDT decision was made to permanently stop clozapine, and the patient readmitted to the medical ward for further management requiring intravenous fluid hydration. The patient's kidney function tests remained within the normal range for the entire duration. His CK normalised 11 days after permanently stopping clozapine with a significant improvement in his physical and mental state.

## 4. Diagnostic Assessment

In this case, the important differential diagnoses considered were sepsis, anticholinergic syndrome, and malignant hyperthermia. Blood cultures revealed no growth of organisms, and the patient never developed hypertension or tachypnoea during this period. Troponin levels were normal. Clinical examination of the chest revealed a clear chest with normal heart sounds. His abdominal examination was normal. Although he did not develop fever, he had other major and minor criteria features as per the DSM-5 criteria [2]. Alternative criteria for diagnosing NMS suggested Levenson's criteria which explained that if a patient had rigidity and elevated CK (2 major symptoms) in addition to tachycardia, abnormal blood pressure, altered consciousness, diaphoresis, or leucocytosis (any 4 minor symptoms), a diagnosis of NMS could be considered [3].

## 5. Investigations and Treatment

The patient was transferred to the medical ward for a thorough physical health evaluation following his injury on the psychiatric unit. He had a computerised tomography (CT) head which was normal. He had blood tests including a creatinine kinase level which was 651 U/L initially. Although mildly elevated, this was attributed to rhabdomyolysis as a result of the injury. There was no leucocytosis noted on the blood test results.

His electrocardiogram revealed sinus tachycardia with a corrected Q-T interval of 432 msec. Lactate was not elevated, and a chest X-ray revealed no abnormalities. His kidney function tests remained normal. Blood cultures were also done as part of the admission protocol, and these yielded no growth. His lumbar puncture results were also normal.

While under the care of the medical team, the patient received intravenous fluid therapy. Benzodiazepines were considered but not clinically indicated due to initial diagnostic uncertainty, and the patient was not agitated or catatonic at the time of presentation to the emergency department.

## 6. Discussion

The pathophysiology of NMS at a receptor level involves a decrease in central dopaminergic activity at the Dopaminergic D2 receptor. This leads to the patient's characteristic physical symptoms which include autonomic dysfunction, muscle rigidity, hyperthermia, and mental state changes. The lack of significant EPS correlates with clozapine's weak affinity for D2 receptors.

A 2015 systematic review highlighted the atypical presentation of NMS with the use of clozapine. Patients presented with autonomic instability and fever but had a lower and delayed CK measurement. They rarely had EPS, and it was concluded that clozapine associated NMS was the most atypical amongst second-generation antipsychotics [1].

Other cases have also revealed the atypical presentation of NMS in patients treated with clozapine. A case report in 2017 described the presentation of a patient diagnosed with NMS. A 55-year-old male, who had been diagnosed with schizophrenia for 10 years and received clozapine, presented with fluctuating consciousness, disorientation, muscle rigidity, and raised CK; however, his temperature was measured 37.2°C. Despite this, he was managed with the assumption of NMS and recovered overtime with a decreasing CK level. This showed an atypical presentation, as the diagnosis would not be achieved with the DSM-5 criteria [4].

Another case report described a 53-year-old female patient diagnosed with NMS. The patient had signs and symptoms of confusion, fever, autonomic instability, and leucocytosis. However, there was an absence of muscle rigidity and a CK of 2158 U/L. This further highlights the possible atypical presentation of NMS with clozapine; however, in this case, the features could be due to clozapine toxicity, as blood tests revealed a toxic level in the patient [5].

Lastly, a review with 11 published cases conducted by Sachdev et al. concluded that although NMS does occur with clozapine, there may be patients who present atypically. NMS with clozapine may have fewer motor signs and a milder CK disturbance [6].

Prior to commencing clozapine, the patient was on paliperidone LAI monthly injections and tolerated this medication without any side effects. The last paliperidone dose was administered 23 days prior to commencing clozapine. Given the median half-life of paliperidone between 25 and 50 days, it may be that his symptoms were due to an interaction between residual paliperidone and clozapine. If Levenson's criteria were applied to this patient, he would have met the threshold for a diagnosis of NMS having 2 core symptoms (rigidity and a rise in CK levels) and 4 minor symptoms (diaphoresis, tachycardia, abnormal blood pressure, and altered consciousness).

## 7. Outcome and Follow-Up

On permanently stopping clozapine, a significant improvement in the patient's physical and mental state was noted. The mild truncal rigidity, diaphoresis, confusion, and CK levels subsided over a period of 2 weeks.

The BMJ Best Practice recommends waiting for at least 2 weeks before reinitiating antipsychotic medication. Since this gentleman had already tried various antipsychotic medications, there were limited options available when ascertaining an alternative antipsychotic medication. Expert opinion from the hospital medicine pharmacy specialist was sought, and they advised rechallenge with oral risperidone as this medication was previously tolerated by the patient. Due to a lack of evidence in the literature pertaining to restarting depot medication after an episode of NMS, the importance of avoiding this was stressed by the pharmacy specialist.

Currently, the patient has been discharged from the psychiatric inpatient unit on risperidone with appropriate safety netting advise and risk management in place. Regular community mental health team reviews and home treatment team follow-up should assist in medication compliance and frequent monitoring of his mental health.

## Additional Points

*Learning Points*. Tachycardia should raise the suspicion of NMS. Diaphoresis is also very common and not often thought of as a hallmark sign of NMS. Elevation of CK is frequent but is not always extreme and may actually be absent in some cases. It may be clinically safe if patients taking clozapine are monitored with Levenson's criteria as this would lower the threshold for investigating NMS. Waiting for rigidity or the development of fever may delay the diagnosis of NMS in patients taking clozapine. In the absence of fever with the presence of other core symptoms of NMS, an atypical presentation should be considered. *Patient Perspective*. Patient or next of kin not wishing to provide any comments.

## References

[1] Belvederi Murri M., Guaglianone A., Bugliani M. (2015). Second-generation antipsychotics and neuroleptic malignant syndrome: systematic review and case report analysis. *Drugs in R and D*.

[2] Simon L. V., Hashmi M. F., Callahan A. L. (2020). *Neuroleptic Malignant syndrome [Internet]*.

[3] Ambulkar R. P., Patil V. P., Moiyadi A. V. (2012). Neuroleptic malignant syndrome: a diagnostic challenge. *Journal of Anaesthesiology Clinical Pharmacology*.

[4] Leonardo Q.-F., Juliana G.-R., Fernando C.-A. J. (2017). Atypical neuroleptic malignant syndrome associated with use of clozapine. *Case Reports in Emergency Medicine*.

[5] Kamış G. Z., Ayhan Y., Basar K., Özer S., Yağcıoğlu A. E. A. (2014). A case of clozapine intoxication presenting with atypical NMS symptoms. *International Journal of Neuropsychopharmacology*.

[6] Sachdev P., Kruk J., Kneebone M., Kissane D. (1995). Clozapine-induced neuroleptic malignant syndrome: review and report of new cases. *Journal of Clinical Psychopharmacology*.

